# A Small Regulatory RNA Contributes to the Preferential Colonization of *Escherichia coli* O157:H7 in the Large Intestine in Response to a Low DNA Concentration

**DOI:** 10.3389/fmicb.2017.00274

**Published:** 2017-02-27

**Authors:** Runhua Han, Letian Xu, Ting Wang, Bin Liu, Lei Wang

**Affiliations:** ^1^TEDA Institute of Biological Sciences and Biotechnology, Nankai UniversityTianjin, China; ^2^The Key Laboratory of Molecular Microbiology and Technology, Ministry of EducationTianjin, China; ^3^Tianjin Key Laboratory of Microbial Functional GenomicsTianjin, China; ^4^State Key Laboratory of Medicinal Chemical Biology, Nankai UniversityTianjin, China

**Keywords:** sRNA, O157:H7, site-preferential colonization, DeoR, DNA concentration

## Abstract

Enterohemorrhagic *Escherichia coli* (EHEC) serotype O157:H7 (O157) is one of the most notorious human pathogens, causing severe disease in humans worldwide. O157 specifically colonizes the large intestine of mammals after passing through the small intestine, and this process is influenced by differential signals between the two regions. Small regulatory RNAs (sRNAs) are able to sense and respond to environmental changes and regulate diverse physiological processes in pathogenic bacteria. Although some sRNAs of O157 have been extensively investigated, whether these molecules can sense differences between the small and large intestine and influence the preferential colonization in the large intestine by O157 remains unknown. In this study, we identified a new sRNA, *Esr055*, in O157 which senses the low DNA concentration in the large intestine and contributes to the preferential colonization of the bacteria in this region. The number of O157 wild-type that adhered to the colon is 30.18 times higher than the number that adhered to the ileum of mice, while the number of the Δ*Esr055* mutant that adhered to the colon decreased to 13.27 times higher than the number adhered to the ileum. Furthermore, we found that the expression of *Esr055* is directly activated by the regulator, DeoR, and its expression is positively affected by DNA, which is significantly more abundant in the ileum than in the colon of mice. Additionally, combining the results of informatics predictions and transcriptomic analysis, we found that several virulence genes are up-regulated in the Δ*Esr055* mutant and five candidate genes (*z0568, z0974, z1356, z1926*, and *z5187*) may be its direct targets.

## Introduction

Small regulatory RNAs (sRNAs) are an emerging class of regulatory molecules in many bacterial species that are typically 50–200 nucleotides in length and do not normally code for protein products (Ryan et al., 2015). Previous studies suggest that these molecules are able to sense and respond to environmental changes rapidly, and that they regulate diverse physiological processes in bacteria including energy metabolism, quorum sensing, stress response, and bacterial virulence (Sharma and Heidrich, 2012; Michaux et al., 2014; Wang et al., 2016). sRNAs act by base-pairing to specific target mRNAs and changing their translation and/or stability, bringing about either repression or activation by exposing the ribosome binding sites or affecting mRNA stability (Gottesman, 2004; Gottesman and Storz, 2011). Many sRNAs require the RNA binding protein, Hfq, as an essential cofactor to regulate mRNA targets, as well as their own stability (Gottesman, 2004; Christiansen et al., 2006). Pathogenic bacteria encounter more diverse environmental conditions than commensals, therefore, their survival can depend on possessing regulatory systems that are able to respond rapidly to changes in their surroundings (Gripenland et al., 2010). Hence, the potential role of sRNAs in bacterial virulence has attracted a great deal of attention. A series of new sRNAs have been identified in different pathogens, some of which have roles both in responding to environmental changes and regulating virulence (Gripenland et al., 2010). For instance, *Ssr1* of *Shigella flexneri* regulates the virulence of this bacteria by mediating its response to acidic environmental changes (Wang et al., 2016).

Previous studies have suggested that virulence factors expressed by gut pathogens represent an energetic burden, which may retard their growth and influence their fitness within the mammalian intestine (Sturm et al., 2011; Pacheco et al., 2012). These data suggest that delicate regulation of the timing and location of the expression of the virulence factors of a pathogen is important for the fitness of the organism. Enterohemorrhagic *Escherichia coli* (EHEC) serotype O157:H7 (referred to herein as O157) is a foodborne agent associated with outbreaks worldwide and poses a serious public health concern (Kaper et al., 2004). O157 causes a wide spectrum of illnesses, ranging from mild diarrhea to severe diseases, such as hemorrhagic colitis and the life-threatening sequelae, hemolytic uremic syndrome (Welinder-Olsson and Kaijser, 2005; Gouali and Weill, 2013). O157 specifically colonizes the large intestine of mammals after passing through the small intestine, which is believed to be the first step of disease development (Torres et al., 2005; Barnett Foster, 2013). The preferential colonization in the large intestine, rather than the small intestine, by O157 is highly regulated in response to different environmental stimuli (Mellies and Lorenzen, 2014). For example, bile salts secreted into the small intestine can serve as an environmental cue to up-regulate the expression of several genes (*acrAB, basRS/pmrAB, arnBCADTEF*) in O157, thereby enhancing its successful migration through the small intestine while at the same time suppressing the expression of virulence factors required for subsequent colonization of the large intestine (Kus et al., 2011). In addition, the low-biotin status in the large intestine promotes the adherence of O157 to epithelial cells, through inducing the expression of locus for enterocyte effacement (LEE) genes which encode a type III secretion system that mediates intimate attachment of bacteria to host cells and the formation of attaching and effacing intestinal lesions (Mellies et al., 2007; Yang et al., 2015).

Over the past decades, hundreds of sRNAs have been identified in *E. coli* by genome-wide searching, using a variety of approaches, and many of these molecules have been well characterized (Majdalani et al., 1998; Masse and Gottesman, 2002; Dubey et al., 2003; Udekwu et al., 2005; Rice and Vanderpool, 2011). However, the majority of these studies were performed in the non-pathogenic *E. coli* strain, K-12, and few investigations have been undertaken in pathogenic *E. coli*, including O157. Recently, several O157 sRNAs, including Hfq-interacting sRNAs, were found within bacteriophage- or O-island-derived genomic regions (Tree et al., 2014; Gruber and Sperandio, 2015). Some of these sRNAs can regulate virulence genes of O157 (Laaberki et al., 2006; Sudo et al., 2014; Tobe et al., 2014; Tree et al., 2014; Gruber and Sperandio, 2015); however, the functions of the majority remain to be elucidated. To date, only ten O157 sRNAs (AsxR, AgvB, Esr41, sRNA56, sRNA103, sRNA350, *arl*, DsrA, GlmY, and GlmZ) have been studied in detail (Laaberki et al., 2006; Sudo et al., 2014; Tree et al., 2014; Gruber and Sperandio, 2015). Overexpression of sRNA350 and DsrA can activate transcription from all LEE-encoded operons by influencing the master regulator *ler* (Laaberki et al., 2006; Gruber and Sperandio, 2015). GlmY and GlmZ destabilize the 3′ fragments of the LEE4 and LEE5 operons, while enhancing translation of the non-LEE-encoded effector, EspFu (Gruber and Sperandio, 2015). AsxR and AgvB act as anti-sRNAs to regulate heme oxygenase and amino acid metabolism, respectively (Tree et al., 2014). However, whether the sRNAs of O157 can respond to environmental signals (e.g., the differences between the small and large intestines) or influence site-preferential colonization remains unknown.

In *E. coli*, DeoR has been identified as a repressor of bacterial deoxynucleoside metabolic genes (*deoCABD*) and nucleoside-transport genes (*nupG* and *tsx*) (Valentin-Hansen et al., 1986; Bremer et al., 1990; Munch-Petersen and Jensen, 1990). This repressor protein binds distant separated operator sites with palindromic sequences in the promoter regions of these genes, generating a DNA loop that is necessary for full repression (Dandanell et al., 1987; Amouyal et al., 1989). Deoxyribose-5-phosphate is a small-molecule effector of DeoR and binding of deoxyribose-5-phosphate to DeoR severely impairs the capacity of DeoR to bind to its cognate operator sites (Dandanell et al., 1987; Amouyal et al., 1989; Mortensen et al., 1989). Deoxyribose-5-phosphate is also an intermediate metabolite of DNA metabolism. In the mammalian gut, DNA is highly abundant, as it is released from eukaryotic cells, foodstuffs, and bacteria (Palchevskiy and Finkel, 2006). *E. coli* can transport these DNA molecules into the cell and consume them as a nutrient, generating deoxyribonucleosides (Finkel and Kolter, 2001). After cleavage of the *N*-glycosidic bond from deoxyribonucleosides by nucleoside phosphorylases (encoded by *deoA* and *deoD*), the pentose moiety, released as deoxyribose-1-phosphate, is isomerized by phosphoribomutase (encoded by *deoB*) into deoxyribose-5-phosphate, which can be split by deoxyriboaldolase (encoded by *deoC*) into acetaldehyde and glyceraldehyde 3-P (Sgarrella et al., 1997; Finkel and Kolter, 2001). However, whether and how DeoR regulates other genes remains unclear.

In this study, we identified a new O157 sRNA, which we refer to as *Esr055* (EHEC small regulatory RNA 055), and investigated its role in the site-preferential colonization in the large intestine of mice by O157. We demonstrated that *Esr055* facilitates preferential adherence of bacteria to the colon of mice. By bioinformatics analysis, chromatin immunoprecipitation (ChIP) experiments, and quantitative real-time polymerase chain reaction (qRT-PCR), we revealed that the expression of *Esr055* is directly activated by DeoR. Additionally, the expression of *Esr055* was positively affected by exogenous DNA, which is more abundant in the small intestine than the large intestine of mice. Finally, we found five potential target genes of *Esr055* and found that several virulence genes, including *stx2*, flagellar, and fimbrial genes, are regulated by this sRNA using transcriptome analysis. This study implies that the regulation of *Esr055*, which is sensitive to exogenous DNA, plays an important role in the site-preferential colonization and virulence of O157.

## Materials and Methods

### Bacterial Strains, Plasmids, and Growth Conditions

Bacterial strains and plasmids used in this study are listed in Supplementary Table S1. EHEC strain EDL933 (serotype O157:H7) and derivatives thereof were cultured in Dulbecco’s Modified Eagle’s Medium (DMEM) or on 1% lysogeny broth (LB) agar unless otherwise specified. When necessary, antibiotics were used at the following concentrations: ampicillin (Sigma), 100 μg/ml; chloramphenicol (Sigma), 15 μg/ml; and kanamycin (Sigma), 50 μg/ml.

### Strain Construction

Mutant strains were generated by substitution of specific genes with a chloramphenicol acetyltransferase (*cat*) gene using the λ Red recombinase system, as described previously (Datsenko and Wanner, 2000). Complementary strains were constructed by cloning genes of interest into the low-copy expression vector, pWSK29 (Wang and Kushner, 1991); PCR products were digested with EcoRI and BamHI, and ligated into EcoRI/BamHI-digested pWSK29. The resulting constructs were then electroporated into the corresponding mutant strains. The *deoR*-3xFLAG strain was constructed by substitution of the *deoR* termination codon with the 3xFLAG epitope and a chloramphenicol resistance cassette amplified from the plasmid, pLW1600 (Ju et al., 1997). For the construction of the *Esr055*-lux translational fusion, a PCR fragment containing nucleotides -114 to +10 relative to the *Esr055* transcription start site was digested with XhoI and BamHI and ligated into XhoI/BamHI-digested pMS402 (Duan et al., 2003). The recombined plasmid was electroporated into the O157 wild-type, the Δ*deoR* mutant, and the *deoR* complementary strain. All strains were verified by PCR amplification and sequencing. The primers used in this study are presented in Supplementary Table S2.

### Small RNA Candidate Region Selection

Given that a large proportion of sRNAs are *trans*-encoded in the intergenic regions (IGR) of the genome, we searched the potential IGR sRNAs of *E. coli* EDL933 using published transcriptome data (Yang et al., 2015). Sequence reads were aligned to the reference sequence of *E. coli* EDL933 using Burrows-Wheeler Aligner software, and coverage plots were produced using SAMtools (Li et al., 2009). Novel intergenic peaks of 50–500 bp in length that formed continuous regions of coverage, with expression distinct from those of flanking open reading frames, were selected by visual inspection using the Artemis genome browser. The sequences of selected peaks were extracted and searched against the Rfam database to eliminate known intergenic sRNAs (Nawrocki et al., 2015). Finally, reads per kilobase per million base pairs (RPKM) values were used to estimate the expression levels of all the resulting sRNA candidates (Trapnell et al., 2009).

### RNA Isolation

*E. coli* EDL933 cells cultured at 37°C were harvested during mid-log phase [optical density measured at a wavelength of 600 nm (OD_600_) value of approximately 0.6] by centrifugation at 12,000 × *g* for 10 min. Total RNA was isolated using TRIzol Reagent (Invitrogen; #15596-018), according to the manufacturer’s protocol. RNA pellets were dissolved in RNase-free H_2_O. Next, RNA samples were treated using a TURBO-DNA free kit (Ambion; #AM1907) to eliminate contaminating genomic DNA. RNA quantity was determined by measuring absorbance at 260 and 280 nm using a NanoDrop-2000 spectrophotometer and the integrity of the RNA was verified by agarose gel electrophoresis.

### 5′ and 3′ Rapid Amplification of cDNA Ends (RACE)

Rapid amplification of cDNA ends (RACE) assays were carried out with the 5′ RACE System for Rapid Amplification of cDNA Ends, Version 2.0 (Invitrogen, #18374-058) and the 3′ RACE System for Rapid Amplification of cDNA Ends (Invitrogen, #18373-019), according to the manufacturer’s protocols. For 5′-RACE, 5 μg of tobacco acid pyrophosphatase (Epicentre Technologies; #RP8092H) treated RNA was reverse transcribed using a sRNA-specific antisense primer and SuperScript^TM^ Reverse Transcriptase Invitrogen; #18091050). cDNA was then purified, dC-tailed, and used as a template in a PCR reaction with the Abridged Anchor Primer (AAP) and a nested gene-specific primer. 3′-RACE was performed by ligating a poly(A) tail, using a Poly(A) Polymerase Tailing Kit (Epicentre; #PAP5104H) before reverse transcription. Specific cDNAs were then directly amplified by PCR using an Anchor Primer (AP) that targets the poly(A) tail region and a gene-specific primer that anneals to a region of known sRNA sequence. PCR products were cloned into the pGEM^®^-T Vector System (Promega; #A3600) before sequencing. Primers used in RACE assays are presented in Supplementary Table S2.

### Northern Blot Assays

Northern blot assays were performed using a previously described method (Mann et al., 2012). Total RNA (5 μg) was separated on a 1.2% agarose gel containing 37% formaldehyde at 120 V for 30 min. Gels were briefly rinsed in 0.5x Tris/borate/ethylenediaminetetraacetic acid (EDTA) buffer and electroblotted onto Brightstar Plus nylon membranes (Applied Biosystems) at 20 V for 40 min, which were then baked at 120°C for 30 min. Crosslinked membranes were prehybridized for 1 h at 50°C in digoxigenin (DIG) Easy Hyb buffer. Oligonucleotide probes were labeled using the DIG Oligonucleotide Tailing Kit (Roche; #03353583910), added to fresh Easy Hyb buffer (10 pmol/mL), and the blots incubated with hybridization buffer overnight at 50°C. After high- and low-stringency washes, blots were further washed using the DIG Wash and Block Buffer Kit (Roche) and then CDP-Star (Roche) was added as the substrate. Hybridization signals were visualized using a Phosphorimager (Molecular Dynamics). The size of each transcript was determined by comparing its corresponding band to the low range ssRNA ladder (New England Biolabs, #N0364S). 5S rRNA was used as an internal control. The specific probes used in the northern blot assays are presented in Supplementary Table S2.

### Cell Adherence Assays

Cell infection assays using human HeLa epithelial cells (ATCC CCL-2) were performed as described previously (Jandu et al., 2009; Kim et al., 2009; Yang et al., 2015). Overnight cultures were grown in DMEM to reach a OD600 of 0.6 at 37°C for adaptation. HeLa cells were washed three times with pre-warmed phosphate-buffered saline (PBS), and the medium replaced with fresh DMEM without antibiotics or fetal bovine serum. Cells were then infected with bacterial culture (Wild-type, Δ*Esr020*, Δ*Esr023*, Δ*Esr025*, Δ*Esr055*, Δ*Esr060*, Δ*Esr077*, Δ*Esr091*, and Δ*Esr096*) in DMEM at a multiplicity of infection of 100:1 (Jandu et al., 2006, 2009). After 3 h of incubation at 37°C in 5% CO_2_, cells were washed three times in pre-warmed PBS to remove unbound bacteria and then lysed in 1 ml 0.1% sodium dodecyl sulfate (SDS). Lysates were collected, diluted, and plated onto LB agar plates to determine numbers of bacterial colony-forming units (CFU). Each experiment was carried out at least five times.

### Mouse Colonization Experiments

Mice virulence assays were performed using a BALB/c mouse model with intact commensal flora, described previously (Mohawk et al., 2010). Mice were provided with food and water *ad libitum*. Five weeks female BALB/c mice (*n* = 7) were administered intragastrically with 100 μl PBS containing 10^9^ bacteria (O157 wild-type and the Δ*Esr055* mutant) growing in logarithmic phase. Infected mice were anesthetized using diethyl ether and euthanized by cervical dislocation 6 h after infection. Colons and ileums were dissected and their luminal contents removed. The processed organs were washed with PBS three times and then weighed and homogenized in cold, sterile PBS. Bacterial CFU values of these samples were determined by plating serially diluted homogenates on LB agar.

### RNA-Seq

The O157 wild-type strain and the Δ*Esr055* mutant were grown to exponential phase in DMEM and total RNA was extracted from three biological replicates, respectively. RNA samples were purified using an RNeasy Mini Kit (Qiagen) and bacterial rRNA was depleted using the Ribo-Zero rRNA Removal Kit (Epicentre; #RZNB1056). Library construction of RNA samples was carried out using a NEBNext^®^ Ultra^TM^ Directional RNA Library Prep Kit for Illumina^®^ (NEB, USA), following the manufacturer’s recommendations. After cluster generation, library preparations were sequenced on an Illumina Hiseq platform to generate paired-end reads. Raw data (raw reads) in fastq format were first processed using in-house Perl scripts, to obtain clean data (clean reads) by removing reads containing adapter or poly-N sequences and low quality reads from the raw data. Clean reads were then mapped to the *E. coli* EDL933 genome using Bowtie2-2.2.3 (Langmead and Salzberg, 2012). HTSeq v0.6.1 was used to count the numbers of reads mapped to each gene. RPKM values were calculated for each gene in each of the samples tested to quantify gene expression levels (Trapnell et al., 2009). Genes showing a twofold or greater difference in RPKM between two conditions were defined as differentially expressed.

### Quantitative Real-Time PCR

RNA was isolated from three independent cultures and treated with DNAase with the TURBO-DNAfree kit (Ambion; #AM1907) prior to reverse transcription. cDNA was generated from 1.2 μg of total RNA using a PrimeScript 1st Strand cDNA Synthesis Kit (Takara; #D6110A) with random primers. qRT-PCR was performed using an Applied Biosystems 7300 Real-Time PCR system and SYBR Green PCR Master Mix (Applied Biosystems; #4367659). cDNA templates were denatured at 95°C for 10 min, followed by 45 cycles of 95°C (30 s), 55°C (30 s), and 72°C (60 s). All data were normalized to levels of the housekeeping gene, 16S rRNA (*rrsH*), and relative expression levels were calculated as fold change values using the 2^-ΔΔ^*^C^*^t^ method (Livak and Schmittgen, 2001). Each experiment was carried out in triplicate.

For transcription analysis during mouse infection, 5–6 weeks old female BALB/c mice (*n* = 3) were infected intragastrically with 10^9^ CFU of O157 wild-type. At 6 h following infection, mice were sacrificed by cervical dislocation, and the ileum and colon were removed aseptically. RNA isolation and qRT-PCR analysis were carried out as described above.

For transcription analysis of *Esr055* after adding exogenous DNA in O157 wild-type, the Δ*deoR* mutant and the *deoR* complementary strains, overnight cultures were grown in DMEM supplemented with 0 or 50 μg/ml purified sonicated salmon sperm DNA to reach a OD600 of 0.6 at 37°C. RNA isolation and qRT-PCR analysis was carried out as described above. All experiments were performed independently three times.

### Measurement of Luciferase Activity

Luciferase activity was measured as previously described (Duan et al., 2003). Reporter strains were grown aerobically at 37°C overnight. Cultures were diluted to an OD_600_ of 0.2 and cultivated for 6 h before use. Cultures were inoculated into parallel wells of black 96-well plates with a transparent bottom. Luciferase activity was measured using a Victor3 Multilabel Plate Reader (Perkin-Elmer, USA). Simultaneously, bacteria were diluted and counted on LB agar plates. Relative luciferase activity values were normalized to CFU values. All experiments were performed independently three times.

### Chromatin Immunoprecipitation Assays

Chromatin immunoprecipitation assays were performed as previously described (Lucchini et al., 2006; Davies et al., 2011). Cells were grown aerobically at 37°C to mid-exponential phase (OD_600_ value of approximately 0.6). Formaldehyde was then added to a final concentration of 1% and incubated at room temperature for 25 min. Glycine was added to a final concentration of 0.5 M and cells incubated for a further 5 min to quench the cross-linking reaction. Cross-linked cells were harvested and washed three times with ice cold Tris-buffered saline. Cells were resuspended in 500 μl lysis buffer [10 mM Tris (pH 7.5), 1 mM EDTA, 100 mM NaCl, 1 mM protease inhibitor cocktail, 1 mg/ml lysozyme, 0.1 mg/ml RNase A] and incubated at 37°C for 30 min. Next, 500 μl immunoprecipitation (IP) buffer [100 mM Tris-HCl (pH 7.5), 200 mM NaCl, 1 mM EDTA, 2% (v/v) Triton X-100, 1 mM phenylmethane sulfonyl fluoride] was added and the lysate was sonicated with 20 cycles of 20 s on/off at 95% amplitude to generate DNA fragments of approximately 500 bp. Cell debris was removed by centrifugation at 12,000 × *g* for 10 min. Supernatants were split into two 500 μl aliquots. ChIP was performed with one aliquot using an anti-3 × FLAG antibody (Sigma-Aldrich; #F1804) and protein A magnetic beads (Invitrogen; #10002D). The other aliquot was treated in the same way, but without the addition of any antibodies, as a negative control, mock-IP sample. After washing, beads were resuspended in 200 μl elution buffer [50 mM Tris-HCl (pH 8.0), 10 mM EDTA, 1% SDS] and eluted at 65°C for 20 min. DNA was purified and recovered by phenol–chloroform extraction and ethanol precipitation. All experiments were performed independently three times.

### Measurement of DNA Concentrations in Mouse Intestine

Five groups of 5–6 weeks old female BALB/c mice (three mice each group) were euthanized, and the luminal contents were obtained. The DNA concentrations in the ileal and colonic contents were quantified by a microfluorometric method, described previously (Cesarone et al., 1979). Aliquots of luminal contents were added to 33258 Hoechst fluorochrome (Sigma Chemical, St. Louis, MO, USA), which was dissolved in water to a final concentration of 1.5 μmol/L. Fluorescent readings were recorded using excitation and emission wavelengths of 360 and 450 nm, respectively, and compared with a standard prepared using calf thymus DNA. To further compare DNA concentrations, 50 μg samples of intestine contents were separated by electrophoresis on a 1.2% agarose gel with ethidium bromide at 120 V for 20 min and visualized by ultraviolet transillumination.

### Ethics Statement

All animal experiments were carried out according to the standards set forth in the Guide for the Care and Use of Laboratory Animals. The experimental protocols were approved by the Institutional Animal Care Committee at Nankai University.

### Statistical Analyses

All data are expressed as means ± standard deviation (SD). Differences between two groups were evaluated using independent-samples *t*-test or Mann–Whitney *U* test. Values of *p* ≤ 0.05, 0.01, or 0.001 were considered to be statistically significant (^∗^), highly significant (^∗∗^), or extremely significant (^∗∗∗^), respectively. Data were analyzed using SPSS 12.0 (SPSS Inc., Chicago, IL, USA) for Windows, and figures were drawn using Origin 8.5 (Origin Lab Corporation).

## Results

### Identification of Potential Virulence-Related sRNAs in O157

**FIGURE 1:**
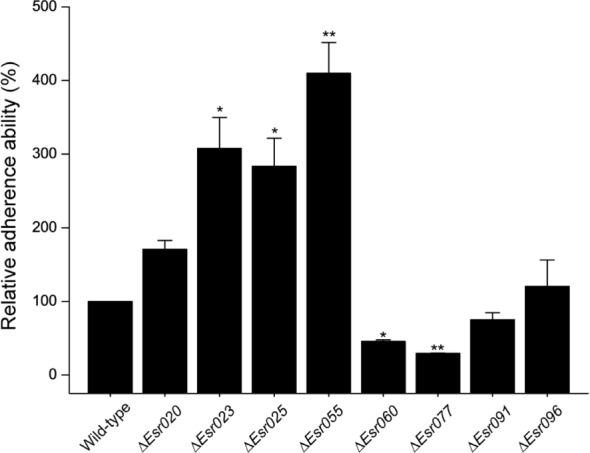
**The adherence of O157 and sRNA mutants to Hela cells.** The relative adherence ability of O157 wild-type was set to 100%, and other values were adjusted accordingly. Data are presented as means ± SD. ^∗^*P* ≤ 0.05; ^∗∗^*P* ≤ 0.01. All *P*-values were calculated using independent-samples *t*-tests.

Through previous transcriptional analysis of the O157 EDL933 strain during infection of Hela cells (Yang et al., 2015), we predicted 96 sRNA candidates in IGR. These sRNAs were termed “*Esr*” (for EHEC small regulatory RNA), and numbered from 001 to 096 (Supplementary Table S3). Among them, eight potential sRNAs (*Esr020, Esr023, Esr025, Esr055, Esr060, Esr077, Esr091*, and *Esr096*) which had not been studied previously and showed the most significant differential regulation in HeLa-attached O157 cells compared with free-living DMEM-grown cells, were selected for further investigation. HeLa infection experiments were carried out as a preliminary comparison of the adherence abilities of wild-type O157 and strains with these sRNAs mutated. We found that the adherence ability of five mutants (Δ*Esr023*, Δ*Esr025*, Δ*Esr055*, Δ*Esr060*, and Δ*Esr077*) was significantly different to that of the wild-type strain (*P* < 0.05) (**Figure 1**). The ability of the Δ*Esr023*, Δ*Esr025*, and Δ*Esr055* mutants to attach HeLa cells were higher than that of the wild-type strain (*P* < 0.05) (**Figure 1**). Conversely, the ability of the Δ*Esr060* and Δ*Esr077* mutants to attach HeLa cells were lower than that of the wild-type strain (*P* < 0.05) (**Figure 1**). No significant changes of the HeLa-attached ability were found between the other three sRNAs mutants (Δ*Esr020*, Δ*Esr091*, and Δ*Esr096*) and the wild-type strain (**Figure 1**). Among these five sRNAs, *Esr055* exhibited the most significant effects on the adherence of O157 and was selected for further study.

### Identification and Characterization of *Esr055* in O157

**FIGURE 2:**
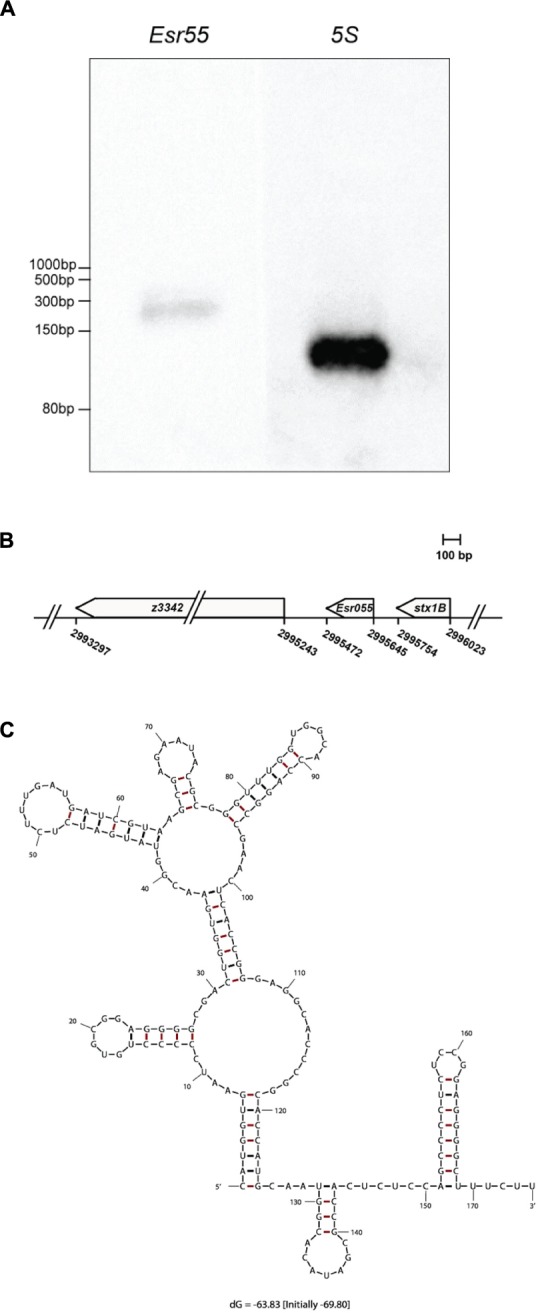
**Identification and characterization of *Esr055*. (A)** The *Esr055* expression of O157 wild-type grown to the logarithmic phase in DMEM; 5S rRNA was used as a control. **(B)** Position of *Esr055* in the O157 genome determined by RACE. **(C)** Secondary structure of *Esr055* predicted using Mfold.

The expression of *Esr055* in O157 was confirmed by northern blot analysis, using 5S rRNA as an internal control. We detected an approximately 170 nt RNA expressed on the minus strand in O157 grown in DMEM at logarithmic phase using a specific probe (**Figure 2A**). The genes flanking *Esr055* on each side are predicted to encode a 9-*O*-acetyl-*N*-acetylneuraminic acid deacetylase (*z3342*) and the B subunit of Shiga toxin 1 (*stx1B*) (**Figure 2B**). To determine the transcription start and termination sites of *Esr055*, we performed 5′ and 3′ RACE. We determined that *Esr055* is 174 nt in length and that the transcription start site (+1) of *Esr055* maps to C_2995645_ in the *E. coli* EDL933 genome, and a putative Rho independent terminator was found at T_2995472_, which is consistent with the northern blot results. The secondary structure of *Esr055* that was predicted using Mfold program (Zuker, 2003) is shown in **Figure 2C**.

### *Esr055* Contributes to the Preferential Colonization of O157 on the Mouse Colon

**FIGURE 3:**
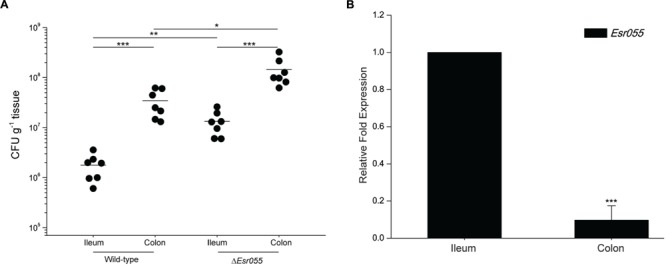
**Contribution of *Esr055* to the virulence of O157 in mice. (A)** The adherence capacity of O157 wild-type strain and the Δ*Esr055* mutant in the intestinal tract of mice. Horizontal lines represent mean values; statistical significance was assessed using a Mann–Whitney *U* test. **(B)** The relative expression of *Esr055* during mouse infection. The relative level of *Esr055* in O157 recovered from ileum was set to 1, and the level in colon was adjusted accordingly. Data are presented as means ± SD. ^∗^*P* ≤ 0.05; ^∗∗^*P* ≤ 0.01; ^∗∗∗^*P* ≤ 0.001. Statistical significance was assessed using independent-samples *t*-test.

To examine the effects of *Esr055* on O157 adherence, we carried out *in vivo* mouse colonization experiments. BALB/c mice were intragastrically administered with 10^9^ CFU of O157 wild-type and the Δ*Esr055* mutant and the number of CFUs recovered from the ileum and colon was determined. The mean number of CFUs recovered from colon infected with the wild-type was 5.24 × 10^7^, which is 30.18-fold higher than that recovered from the ileum (1.74 × 10^6^) (*P* < 0.001) (**Figure 3A**). In contrast, the mean CFU value recovered from colon infected with the Δ*Esr055* mutant (1.72 × 10^8^) was 13.27-fold higher than that recovered from the ileum (1.29 × 10^7^) (*P* < 0.001). The numbers of adhered Δ*Esr055* mutant in both the ileum (7.41 times and 3.28 times, respectively) and colon were significantly higher than those of the wild-type strain (*P* < 0.05) (**Figure 3A**). Additionally, we monitored the *Esr055* level in mouse intestine by qRT-PCR analysis. The expression of *Esr055* in the colon was 10.56-fold lower than that in the ileum (*P* < 0.001) (**Figure 3B**). These results indicated that *Esr055* is a negative regulator of O157 adherence and benefits its preferential colonization in the colon.

### Regulation of *Esr055* by DeoR

**FIGURE 4:**
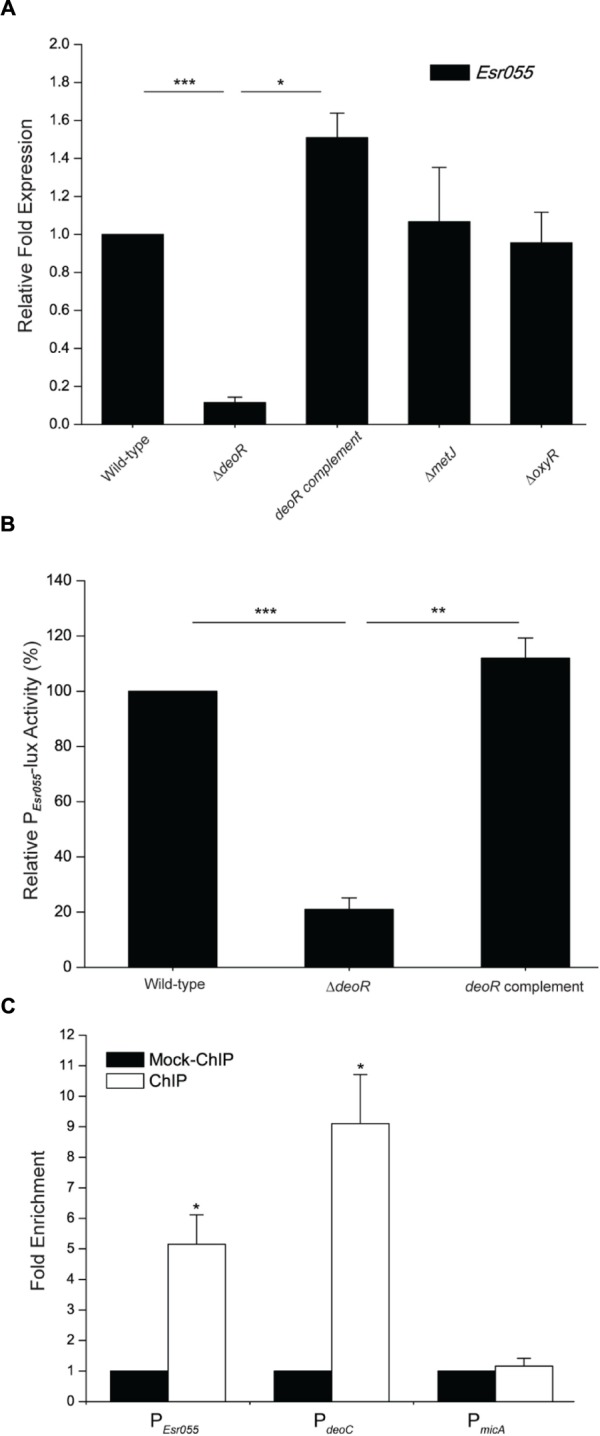
**DeoR regulates the expression of *Esr055* by binding to its promoter. (A)** The relative expression of *Esr055* in O157 wild-type, the Δ*deoR* mutant, the *deoR* complementary strain, the Δ*metJ* mutant, and the Δ*oxyR* mutant. **(B)** The relative activity of P*_Esr055_*-lux in O157 wild-type, the Δ*deoR* mutant, and the *deoR* complementary strains. The relative activity of P*_Esr055_*-lux in the wild-type strain was set to 100%, and the other values were adjusted accordingly. **(C)** Fold enrichment of the *Esr055* promoter in O157 ChIP samples, as determined by ChIP-qPCR. *deoC* and *micA* were positive and negative controls, respectively. Data are presented as means ± SD. ^∗^*P* ≤ 0.05; ^∗∗^*P* ≤ 0.01; ^∗∗∗^*P* ≤ 0.001. All *P*-values were calculated using independent-samples *t*-tests.

Examination of the upstream region of *Esr055* revealed putative OxyR (TAGAATAG), DeoR (TTAGAATA), and MetJ (ATACATCT) binding sites. Inactivation of *deoR* in O157 resulted in a significant reduction of the expression of *Esr055* (*P* < 0.001), and complementation of the Δ*deoR* mutant with *deoR* restored the *Esr055* expression level to that observed in the wild-type strain (*P* < 0.05) (**Figure 4A**). No significant changes of *Esr055* expression were found between the Δ*oxyR* or Δ*metJ* mutants and the wild-type (**Figure 4A**). We then constructed an *Esr055* promoter-lux fusion (P*_Esr055_*-lux) and measured its activity in wild-type, the Δ*deoR* mutant, and the *deoR* complementary strain. The activity of P*_Esr055_*-lux was 4.77-fold lower in the Δ*deoR* mutant compared with that in the wild-type (*P* < 0.001) (**Figure 4B**). Moreover, the complementation of the Δ*deoR* mutant with *deoR* fully restored P*_Esr055_*-lux activity to wild-type levels (*P* < 0.01) (**Figure 4B**). ChIP-qPCR analysis demonstrated that DeoR binds to the promoters of *Esr055* and *deoC* (*P* < 0.05) (positive control), but not to the promoter of *micA* [negative control, which is exclusively transcribed by σ^E^ (Udekwu and Wagner, 2007)] (**Figure 4C**). Taken together, our results demonstrate that DeoR regulates the expression of *Esr055* through direct binding to its promoter region.

### Different DNA Concentrations Effect the Expression of *Esr055* via DeoR

**FIGURE 5:**
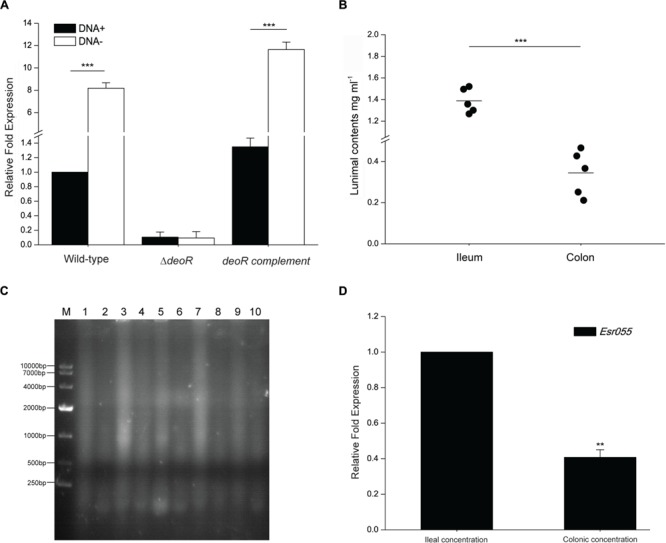
**The effect of exogenous DNA on *Esr055* expression. (A)** The relative expression of *Esr055* in O157 wild-type, the Δ*deoR* mutant and the *deoR* complementary strains grown in DMEM supplemented with 0 (DNA-) or 50 μg/ml (DNA+) purified sonicated salmon sperm DNA. **(B)** DNA concentrations in the ileal and colonic contents of BALB/c mice. Each data point represents a group of three mice. Horizontal lines represent mean values. Statistical significance was assessed using a Mann–Whitney *U* test. **(C)** Electrophoresis of 50 μg of the ileal and colonic contents of BALB/c mice. M: DL10000 DNA marker, Lanes 1, 3, 5, 7, 9: samples of the ileal contents from each group of three mice; Lanes 2, 4, 6, 8, 10: samples of the colonic contents from each group of three mice. **(D)** The relative expression of *Esr055* in the presence of DNA concentrations simulating those in the ileal and colonic contents. The relative level of *Esr055* in the presence of 1.39 mg/ml DNA (ileal concentration: simulating the DNA concentration in the ileal contents) was set to 1, and the level of *Esr055* in the presence of 0.34 mg/ml DNA (colonic concentration: simulating the DNA concentration in the colonic contents) was adjusted accordingly. Data are presented as means ± SD. ^∗∗∗^*P* ≤ 0.001. Statistical significance was assessed using independent-samples *t*-tests.

The regulation of DeoR is influenced by deoxyribose-5-phosphate (Dandanell et al., 1987; Amouyal et al., 1989; Mortensen et al., 1989), which is also an intermediate product of DNA metabolism (Sgarrella et al., 1997; Finkel and Kolter, 2001); therefore, we carried out RT-PCR analysis to examine the effect of DNA on *Esr055* expression. The results showed that the expression of *Esr055* was strongly induced when wild-type cells were exposed to sonicated exogenous DNA (**Figure 5A**). In contrast, the expression of *Esr055* was not affected by DNA in the Δ*deoR* mutant (*P* < 0.001) (**Figure 5A**). The regulation of *Esr055* by DNA could be restored in the *deoR* complementary strain (*P* < 0.001) (**Figure 5A**).

We next measured the DNA concentration in the intestine of uninfected mice and found that the concentration of total DNA in the ileal contents is 1.39 mg/ml, which is significantly higher than that in the colonic contents (0.34 mg/ml) (*P* < 0.001) (**Figure 5B**). Gel electrophoresis also demonstrated a higher DNA level in the ileal contents than that in the colonic contents (**Figure 5C**). qRT-PCR analysis was then carried out to examine the expression of *Esr055* in the presence of concentrations of DNA resembling those detected in ileum and colon. The expression of *Esr055* was significantly decreased in the presence of 1.39 mg/ml (simulates the concentration in the colonic contents), compared with 0.34 mg/ml (simulates the concentration in the ileal contents) DNA (*P* < 0.01) (**Figure 5D**).

### Identification of Potential Targets of *Esr055*

Small regulatory RNAs usually repress their targets at the post-transcriptional level by direct interaction with mRNA molecules (Gottesman, 2004; Sharma and Heidrich, 2012). To identify the targets of *Esr055*, we used two independent programs, TargetRNA2 and IntaRNA (Busch et al., 2008; Kery et al., 2014) to search the 5′ regions (approximately -120 to +20 nt relative to the start codon) of O157 mRNAs for potential RNA duplex formation sequences. This analysis predicted a total of 121 potential targets with *P*-values ≤ 0.05, suggesting that *Esr055* may interact directly with these target mRNAs. Transcriptome sequencing was subsequently performed to confirm these targets and detect differences in global gene expression profiles between O157 wild-type and the Δ*Esr055* mutant. In total, 19,188,145 and 17,675,095 raw reads were generated using the Illumina RNA-seq technique in O157 wild-type and the Δ*Esr055* mutant, respectively. After low quality reads were removed, 15,043,159 and 13,788,967 reads from each library were successfully mapped to the *E. coli* EDL933 genome, with gene coverage levels of 99.43 and 99.07%, respectively. A total of 5,100 genes were detected in O157 wild-type and 5,125 in the Δ*Esr055* mutant, and those differentially expressed (≥2-fold) between the two samples were selected for further analysis.

**FIGURE 6:**
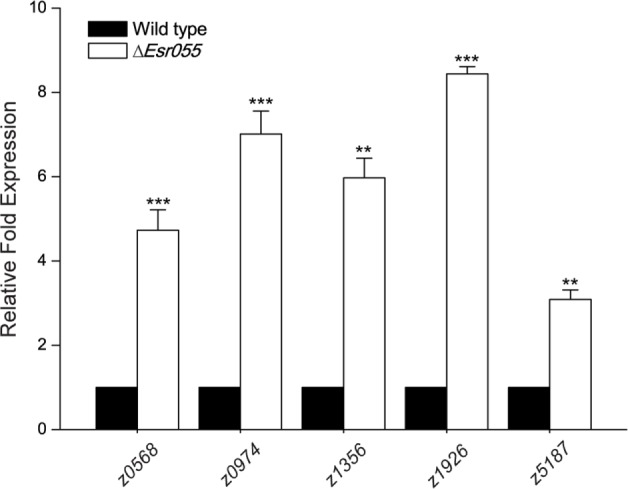
**qRT-PCR of five mRNA targets of *Esr055*.** The relative expression of *z0568, z0974, z1356, z1926*, and *z5187* in O157 wild-type and the Δ*Esr055* mutant. Data are presented as means ± SD. ^∗∗^*P* ≤ 0.01; ^∗∗∗^*P* ≤ 0.001. All *P*-values were calculated using independent-samples *t*-tests.

A total 418 genes differentially expressed between O157 wild-type and the Δ*Esr055* mutant were identified, representing 19.8% of the total number of genes in the *E. coli* EDL933 genome. Among them, the 205 candidate genes were up-regulated and 213 down-regulated in the Δ*Esr055* mutant compared with the wild-type (Supplementary Table S4). Fifteen genes (five up-regulated, five down-regulated, and five equally expressed genes) were randomly chosen for validation of the transcriptome results using qRT-PCR assays. Fold-change values obtained using transcriptome and qRT-PCR assays demonstrated a good concordance between the datasets (Supplementary Table S5), implying the reliability of our data. Genes up-regulated in the Δ*Esr055* mutant included: two toxin genes (*stx2B* and *hlyE*); genes involved in the biosynthesis of flagella, fimbria, and colanic acid; and four type III secretion system 2 (ETT2) genes; as well as 130 hypothetical genes and genes of undefined function. The down-regulated genes in the Δ*Esr055* mutant included: genes involved in oxidative phosphorylation, purine metabolism, and periplasmic nitrate reductase; genes involved in the synthesis and/or transport of methyl-galactoside, maltose, phosphate, nickel, dipeptide and lysine; and four transcriptional regulators; as well as 84 hypothetical genes and genes of undefined function. The downregulation of *stx2*, flagella genes, and fimbriae genes were also vertified by qRT-PCR (Supplementary Table S5). The transcriptome data indicates that *Esr055* is involved in multiple physiological processes in O157 and has a particularly essential role in pathogenesis regulation via repression of a numbers of virulence genes. Additionally, by integrating the results of bioinformatic predictions and transcriptome analysis, we found that five mRNAs (*z0568, z0974, z1356, z1926, z5187*) maybe direct targets of *Esr055*. The regulation of these targets by *Esr055* indicated by the transcriptome data was further confirmed by qRT-PCR (**Figure 6**).

## Discussion

In this study, we found eight sRNAs in O157 which are unidentified previously and showed significant differences in gene expression in HeLa-attached, compared with free-living DMEM-grown, O157 cells. Further, we demonstrated that one of these sRNAs, *Esr055*, contributes to the preferential colonization in the large intestine by O157. We found that the DeoR regulator directly activates the expression of *Esr055* by binding to its promoter region, and that the expression of *Esr055* is sensitive to the low DNA concentration in colonic contents of mouse. In addition, we compared the transcriptome profiles between O157 wild-type and the Δ*Esr055* mutant, and found that *Esr055* negatively regulates several virulence-related genes and that five mRNAs (*z0568, z0974, z1356, z1926*, and *z5187*) may be direct targets of *Esr055*, however, whether and how these potential target genes contribute to the virulence of O157 requires further investigation.

Pathogenic bacteria require virulence factors to enhance their fitness and virulence within their hosts (Kaper et al., 2004), however, the pathogen must pay a significant price for the expression of such factors, as this can lead to retardation of their growth and decreased fitness (Sturm et al., 2011; Pacheco et al., 2012). O157 exhibits a site-preferential colonization ability on the large intestine and must successfully survive through the small intestine before approaching the site of colonization and infection. This process requires the precise regulation of virulence factors to facilitate adaption to different microenvironmental cues (Pacheco et al., 2012; Barnett Foster, 2013; Yang et al., 2015). LEE and *stx2* genes are downregulated in O157 in non-pathogenic sites to enhance survival and prevent superfluous energy expenditure (Pacheco et al., 2012; Yang et al., 2015). In the present paper, we demonstrated for the first time that the sRNA, *Esr055*, is involved in regulation of the preferential colonization of O157 in the large intestine (**Figure 3A**). The expression of *Esr055* in the ileum suppresses a number of virulence factors (e.g., *stx2*, flagella genes, and fimbriae genes), hence avoiding unnecessary energy consumption in this non-pathogenic site. Shiga-like toxin is a major cytotoxin of O157 which is responsible for hemolytic uremic syndrome, and also promotes the colonization capacity of O157 by enhancing the expression of nucleolin, a surface-localized intimin receptor (Robinson et al., 2006). Flagella and fimbriae are two other important adhesive appendages in the initial phases of O157 colonization (Musken et al., 2008; Mahajan et al., 2009; Carter et al., 2016). Once entering the colonization site, *Esr055* expression levels decrease sharply (**Figure 3B**), resulting in the derepression of these virulence factors to promote O157 colonization and virulence. While the effects of these virulence genes on O157 colonization have been fully elucidated, how they are regulated by *Esr055* remains unclear.

DeoR has been clearly identified as a repressor of the *deo* operon, *nupG*, and *tsx*, through interaction with palindromic sequences in their promoter regions (Valentin-Hansen et al., 1986; Bremer et al., 1990; Munch-Petersen and Jensen, 1990). In this study, we demonstrated that DeoR is also able to bind the promoter region of *Esr055* (**Figures 4B,C**), which contains an 8 bp sequence (TTAGAA-TA) which partially overlaps with DeoR-binding palindrome sequence (TGTTAGAA-TACTAACA or TGTTAGAA-TTCTAACA) identified in previous studies (Valentin-Hansen et al., 1986; Dandanell et al., 1987; Amouyal et al., 1989), indicating that it is a regulator of *Esr055* expression. However, we found that the expression of *Esr055* was significantly decreased in the Δ*deoR* mutant (**Figure 4A**), suggesting that DeoR acts as an activator of *Esr055* expression, which is differs from its known function as a repressor. Such dual regulatory functions have also been described for several other well-studied regulators, including Fis, Fnr, and PhoP (Zhang X. et al., 2016; Zhang Y.X. et al., 2016; Zheng et al., 2016). Previous studies have also shown that the binding of DeoR to the *deoC* promoter sequence can be hindered by the addition of deoxyribose-5-phosphate, an intermediate product of DNA metabolism (Mortensen et al., 1989), indicating that deoxyribose-5-phosphate can inhibit the function of DeoR. Consistent with these findings, our results indicate that deoxyribose-5-phosphate can also inhibit the regulation of *Esr055* by DeoR, as the expression of *Esr055* was significantly increased in the Δ*deoB* mutant, in which deoxyribose-5-phosphate cannot be synthesized (Supplementary Figure S1). Surprisingly, our results demonstrated that the expression of *Esr055* was up-regulated in a DeoR-dependent manner by addition of exogenous DNA (**Figure 5A**), which induced the expression of the *deoB* gene and thus resulted in the increased production of deoxyribose-5-phosphate (Supplementary Figure S2). Furthermore, the addition of exogenous DNA could still significantly increase the expression of *Esr055* in the Δ*deoB* mutant (Supplementary Figure S3). Thus, we propose that exogenous DNA upregulates the expression of *Esr055* using other pathways except for the pathway that mediated by *deoB*, which will be the subject of future study.

Comparison of transcriptome data from O157 wild-type and the Δ*Esr055* mutant indicated that *Esr055* affects many other genes involved in various physiological pathways (including oxidative phosphorylation, purine metabolism, and transportation of nutrients) in addition to virulence genes (Supplementary Table S4), suggesting that this sRNA may exert other functions in addition to its role in virulence regulation. Combined analyses of transcriptomic data and informatics predictions led to the identification of five genes (*z0568, z0974, z1356, z1926*, and *z5187*) that are predicted to be direct binding targets of *Esr055* (**Figure 6**). Unfortunately, these genes are annotated as encoding hypothetical proteins and their functions remain unknown. Thus, our further study will focus on whether and how these five genes affect the virulence of O157.

## Author Contributions

LW conceived and designed the experiments. RH, LX, and TW performed the experiments and analyzed the data. LW, BL, RH, and LX wrote the paper.

## Conflict of Interest Statement

The authors declare that the research was conducted in the absence of any commercial or financial relationships that could be construed as a potential conflict of interest.
