# Supplementary material for: A Small Regulatory RNA Contributes to the Preferential Colonization of Escherichia coli O157:H7 in the Large Intestine in Response to a Low DNA Concentration
Source: Front Microbiol. 2017 Feb 27;8:274. doi: 10.3389/fmicb.2017.00274 (PMC5326754; doi:10.3389/fmicb.2017.00274)
Supplement: Supplementary file 1 [file Data_Sheet_1.PDF]

A Small Regulatory RNA Contributes to the Preferential Colonization of *Escherichia coli* O157:H7 in the Large Intestine in Response to a Low DNA Concentration

Runhua Han[1,2†], Letian Xu[1,2,3†], Ting Wang[1,2], Bin Liu[1,2,3], Lei Wang[1,2,3,4*]

[1]TEDA Institute of Biological Sciences and Biotechnology, Nankai University, Tianjin, China

[2]The Key Laboratory of Molecular Microbiology and Technology, Ministry of Education, Tianjin, China

[3]Tianjin Key Laboratory of Microbial Functional Genomics, Tianjin, China

[4]State Key Laboratory of Medicinal Chemical Biology, Nankai University, Tianjin, China

[†]These authors contributed equally to the study.

*** Correspondence:** wanglei@nankai.edu.cn

Number of supplementary figures/tables: 8

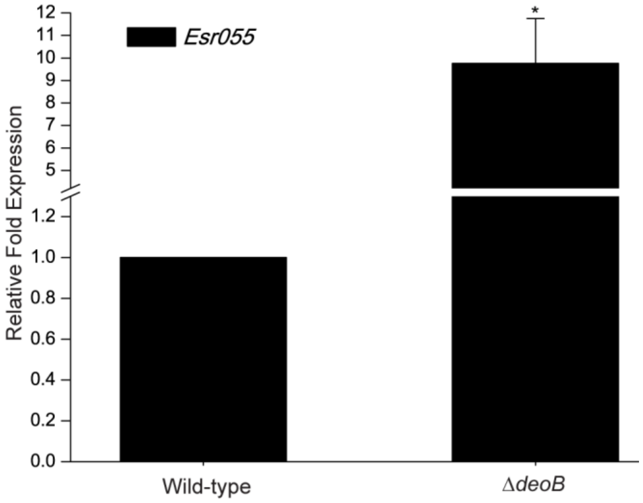

Supplementary Figure S1: The effect of *deoB* on the expression of *Esr055*. The relative expression of *Esr055* in O157 wild-type and the Δ*deoB* mutant. Data are presented as means ± SD. *P ≤ 0.05. All P values were calculated using independent-samples t-tests.

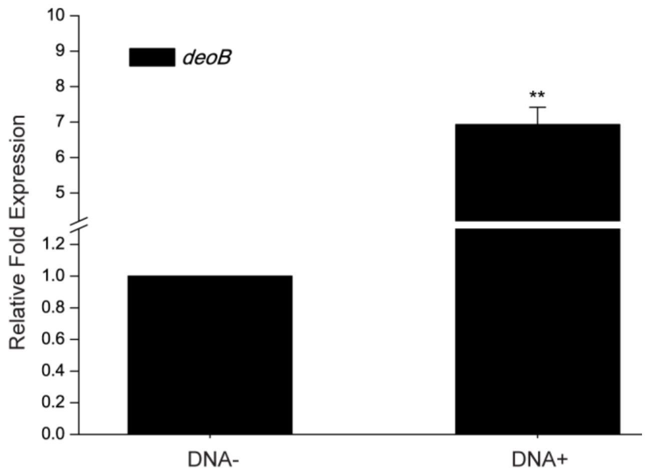

Supplementary Figure S2: The effect of exogenous DNA on the expression of *deoB*. The relative expression of *deoB* in O157 wild-type grown in DMEM supplemented with 0 (DNA-) or 50 µg/ml (DNA+) purified sonicated salmon sperm DNA. Data are presented as means ± SD; n = 3. $^{**}P \leq 0.01$. All P values were calculated using independent-samples t-tests.

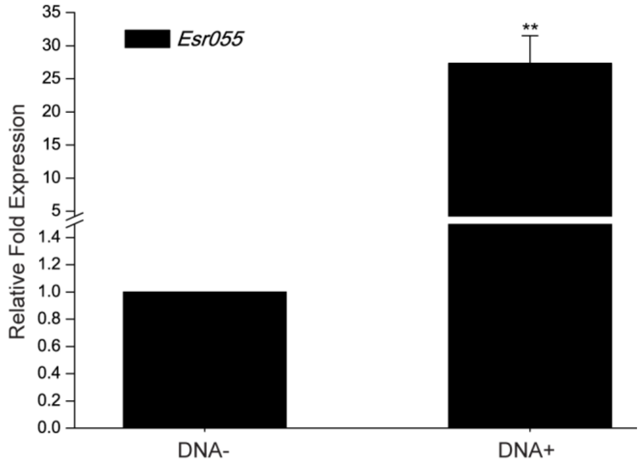

Supplementary Figure S3: The effect of *deoB* on the expression of *Esr55*. The relative expression of *Esr55* in the Δ*deoB* mutant grown in DMEM supplemented with 0 (DNA-) or 50 µg/ml (DNA+) purified sonicated salmon sperm DNA. Data are presented as means ± SD. **P ≤ 0.01. All P values were calculated using independent-samples t-tests.

Supplementary Table S1: Bacterial strains and plasmids used in this study

	**Genotype or description**	**Source or reference**
**Strains**		
G2734	Wild-type EHEC O157:H7 EDL933	ATCC[a]
H2961	*Esr055* deletion mutant in G2734	This study
H2962	*deoR* deletion mutant in G2734	This study
H2963	H2962 containing pLW1878	This study
H2964	G2734 containing pLW1879	This study
H2965	H2962 containing pLW1879	This study
H2966	H2963 containing pLW1879	This study
**Plasmids**		
pKD3	Containing a chloramphenicol resistance cassette and the flipase recognition sites, CmR	(Datsenko and Wanner, 2000)
pKD46	Red recombination plasmid, ApR	(Datsenko and Wanner, 2000)
pWSK29	Expression vector, ApR	(Wang and Kushner, 1991)
pMS402	lux-based promoter reporter plasmid, KmR	(Duan et al., 2003)
pLW1600	pGEM-Teasy carrying 3*FLAG-*cat*, ApR, CmR	(Ju et al., 1997)
pLW1878	pWSK29 carrying *deoR*, ApR	This study
pLW1879	pMS402 containing *Esr055* promoter region	This study

[a]ATCC, American Type Culture Collection, Manassas, Virginia, USA.

Supplementary Table S2: Primers used in this study

Primer	Sequence (5’-3’)
**Gene mutation or replacement**	
Esr055-F	ATAGATGTGTTAGAAAATTTCTGCATGGTGAAT<br>CCCCCTGTGTAGGCTGGAGCTGCTTC
Esr055-R	TTTTGTCCATAAGAAAGCCCCTCCGGAGAGGG<br>GCTGGAGATGGGAATTAGCCATGGTCC
deoR-F	TTAATACATCAACTTAATACGCTGCGTCTGCGC<br>GTACTTGTGTAGGCTGGAGCTGCTTC
deoR-R	ATGGAAACACGTCGCGAAGAGCGTATCGGGCA<br>GCTGCTGATGGGAATTAGCCATGGTCC
deoR-3×FLAG-F	GTATTGAGCGGCTCGCTTCAATAACTATTCAGA<br>GGGATTTTACGCCCCGCCCTGCCACTCA
deoR-3×FLAG-R	AACCGGATGGCGCGAAACGTCATCCGGTTATA<br>CGTCATTGACTACAAAGACCATGACGG
**Gene cloning**	
deoR-F	CCGGAATTCATGGAAACACGTCGCGAA
deoR-R	CGCGGATCCTTAATACATCAACTTAATACGCT
P_{Esr055}-lux-F	CCGCTCGAGCGTTGACTTAGAATAGCTCAG
P_{Esr055}-lux-R	CGCGGATCCATTCACCATGCAGAAATT
**qRT-PCR**	
rrsH-F	GAAAGCGTGGGGAGCAAAC
rrsH-R	ACATGCTCCACCGCTTGTG
Esr055-F	TGATCTCTTTGATGATCGTAAGC
Esr055-R	GCTGGAGAGTGGCGCTATGTGC
z0690-F	CCTGGGCTACTGTTTGTGCA
z0690-R	TGTATAATTCACCAGCGTGCCG
z1465-F	CAGTGAAGGTTGACGGGAAAG
z1465-R	GTACTGGATTTGATTGTGACAG
z2804-F	CCCGGGAATCCTGGAGACT
z2804-R	TGGTGTACAGCCAATCTTTGGG
z3222-F	CCCACCACCAGCGCGTTGTCC
z3222-R	CCACACATTGGCAACGGCGTCG
z4802-F	CAAACCCCAAATCCGGTTTG
z4802-R	TGGTATCAGTGCATTTGCTTAC
z0191-F	GCGATATCGTCATCACCGGCG
z0191-R	CCATGCGCGCGTAAGTCAGGT

z2284-F	CTTACGGATGCCACTACGCTTC
z2284-R	CGGTGATGATCACCAAGCATT
z3586-F	CCGAAGCCGAAGCTGTTAGACA
z3586-R	TCACTCTCTGGGCGCTGCT
z5052-F	CGTTCAAGATCAATGCGATCT
z5052-R	CTGAAGATCTGAAGGATCAAT
z6001-F	GGTCTACATGTTGCAGCAGGA
z6001-R	CCGGATGGAGCGCCAGTAACT
z0535-F	GTGGCTCATGTCCATGCCTTCC
z0535-R	CTGGCCGCGCCTAGCGAAT
z1992-F	CGAAAAGCGACCTGCCGGA
z1992-R	CATCATCCCCTTTGGCATATT
z3413-F	ATCGTTCTGCGGGAACTTCATT
z3413-R	ATCTGCCGTACCGTTCAGGTTT
z4959-F	GATCGAGCGCGTCACGCAGACC
z4959-R	CTCTAACGCCATTCTCGATATG
z5674-F	GGCTTCTCACGCTACTGCTGT
z5674-R	CCACCGGCGCGTTGGATTCC
z0568-F	GTGGTTGCCGTTCCTGCCGATA
z0568-R	AAGGGTATTCAATCCAGCTA
z0974-F	CTGGCGGCGGATGCTGTCGGA
z0974-R	CAAGATGCATCAGTTCTTCGTC
z1356-F	TGGCAGTGCCCGGGTTTCCCTC
z1356-R	TACCACTCGCCGCTTCACTCGC
z1926-F	GCATAGCGATATTGAAATAG
z1926-R	CGATTCAACAATACCCGATAT
z5187-F	TCCCACTGGCGCTGATGCTT
z5187-R	GGATCATTGAGCATCTTAAC
z0688-F	GTTATTGATGCTCATTATA
z0688-R	TTGACCAGAAAACGCTCATTG
z0691-F	ATGAGAAGAGTATTCTTTAGC
z0691-R	TCAATATCCGCTGTGTTTACG
z1708-F	GCCGCGATGGGTTGTTGAGGT
z1708-R	ATTAGTCAACGCTGGCAGGAA
z1712-F	CACTGCTGAATATTTTTGATA
z1712-R	ACCTGGTGCAGCGTTAACCTG
z1713-F	TCCATTGCGGTAACCACCACC
z1713-R	CAATTGCGACGTCAGCTCGTT
z1714-F	TGGCCTTTTCTCAAGCGGTTA
z1714-R	GATAGCAACGTCCAGACCACG
z1717-F	ATGCAAAAAAACGCTGCGCAT
z1717-R	CGTATCGCCAATATTGCGTGG

z3596-F	ACCGTCATTGTGGCGGTGAAA
z3596-R	ATTGATTCGTCATCTTCTGCAA
z3597-F	ACGGACAGCGGTATCGCAGGT
z3597-R	AAACGGATCTCTCTCTTAATGT
z5914-F	TCAATGTAAGGAAATCGCAGG
z5914-R	ACGACCATCCTTTACACCATCG
z5917-F	ATGGCGCAAACGTGGGTATT
z5917-R	CACGCCGCCATAAGCCGAG
**ChIP-qPCR**	
Esr055-promoter-F	CAAATTACGGGGATGACTGTAAC
Esr055-promoter-R	AGTCGCCCCTCCGCACA
deoC-promoter-F	CTGATGCGTTTGCCACCACT
deoC-promoter-R	TGCTTGCTTTCAGATCAGTCA
micA-promoter-F	TGATCGACTGTGAAGCTATCTAA
micA-promoter-R	CACGCCTGACAGAAAAGAAAAA
**Northern blot probe**	
Esr055-p	CGCTTACGATCATCAAAGAGATCATACCGTTCA CCAGTCGCCCCTCCGCA
5S-p	TGAGTTCGGCATGGGGTCAGGTGGGACCACCG CGCTAAGGCCGCCAGGC
**RACE**	
Esr055-5'RACE primer 1	TCCGGAGAGGGGCTGGAGAGT
Esr055-5'RACE primer 2	GAGAGTGGCGCTATGTGCCATTG
Esr055-5'RACE primer 3	TGTGCCATTGCATGGTGCCG
Esr055-3'RACE primer 1	GTTTGGTGGCACCAGGCCGAACT
Esr055-3'RACE primer 2	GGAGGCACCCGGCACCATG

Supplementary Table S3: sRNA candidates in intergenic regions

Name	Start	End	Flanked genes	Length	Fold_change[a]
Esr001	301076	301185	*intH/z0308*	109	0.78
Esr002	301825	301946	*z0308/z0309*	121	1.04
Esr003	902866	903029	*z0958/z0960*	163	1.53
Esr004	903224	903301	*z0958/z0961*	77	1.87
Esr005	904936	905130	*z0961/z0962*	194	0.26
Esr006	963576	963761	*ybiC/ybiJ*	185	0.22
Esr007	1085799	1085866	*z1153/z1154*	67	0.69
Esr008	1087209	1087423	*z1155/z1156*	214	2.4
Esr009	1106381	1106471	*terF/z1178*	90	0.46
Esr010	1139720	1139896	*z1214/z1215*	176	0.74
Esr011	1250318	1250373	*yccA/z1323*	55	0.88
Esr012	1267551	1267741	*z1348/z1349*	190	0.70
Esr013	1325601	1325719	*torD/yccD*	118	0.46
Esr014	1353635	1353804	*stx2B/z1466*	169	1.86
Esr015	1353812	1353974	*stx2B/z1466*	162	1.74
Esr016	1376316	1376697	*lomW/z1490*	381	2.48
Esr017	1388363	1388656	*z1495/z1498*	293	0.34
Esr018	1391960	1392135	*z1503/z1504*	175	1.09
Esr019	1501988	1502079	*ter_F2/z1617*	91	0.48
Esr020	1535325	1535500	*z1654/z1655*	175	12.57
Esr021	1629504	1629616	*z1769/z1770*	112	0.33
Esr022	1641353	1641507	*z1789/z1793*	154	0.59
Esr023	1641514	1641693	*z1789/z1793*	179	0.03
Esr024	1681267	1681343	*z1839/z1840*	76	1.33
Esr025	1866254	1866426	*z2060/z2065*	75	0.07
Esr026	1866433	1866601	*z2060/z2065*	168	0.60
Esr027	1881517	1881619	*z2087/z2088*	102	0.47
Esr028	1888530	1888687	*z2098/z2099*	172	1.04
Esr029	1894005	1894161	*z2107/z2108*	156	1.84
Esr030	1894167	1894227	*z2107/z2108*	60	7.83
Esr031	1903905	1904005	*z2119/z2120*	100	0.31
Esr032	1939632	1939767	*ydeH/ftrA*	135	1.31
Esr033	1939752	1939995	*ydeH/ftrA*	243	4.76
Esr034	2055464	2055642	*z2274/z2275*	178	0.46
Esr035	2138829	2139042	*z2366/z2367*	213	0.49
Esr036	2145322	2145381	*z2377/z2378*	59	0.18

Esr037	2145389	2145544	*z2377/z2378*	155	1.00
Esr038	2283712	2283892	*z6022/z6024*	180	0.36
Esr039	2309581	2309788	*z6048/z6049*	207	1.13
Esr040	2314771	2314962	*z6054/z6055*	191	1.12
Esr041	2324793	2324910	*z6074/z6075*	117	2.84
Esr042	2325301	2325378	*z6075/z6076*	77	0.75
Esr043	2346075	2346192	*ynfM/asr*	117	1.54
Esr044	2412329	2412463	*z2679/purR*	134	0.97
Esr045	2555691	2555774	*z2836/yeaQ*	83	0.60
Esr046	2664225	2664375	*yecI/z2959*	150	0.37
Esr047	2668172	2668237	*yecA/intT*	65	1.64
Esr048	2673712	2673863	*z2975/z2976*	151	0.66
Esr049	2773127	2773319	*z3107/z3108*	192	2.16
Esr050	2815170	2815461	*z3156/z3159*	291	0.66
Esr051	2858716	2858843	*galF/z3206*	127	0.32
Esr052	2898664	2898770	*z3241/z3242*	106	0.17
Esr053	2990283	2990400	*z3335/z3336*	117	0.40
Esr054	2995286	2995465	*z3342/stx1B*	179	0.80
Esr055	2995472	2995645	*z3342/stx1B*	173	0.09
Esr056	2997206	2997291	*stx1A/z3345*	85	0.80
Esr057	3121118	3121212	*rcsB/rcsC*	94	0.25
Esr058	3189158	3189274	*nuoM/nuoL*	116	0.28
Esr059	3236611	3236715	*yfcJ/fabB*	104	2.66
Esr060	3376674	3377013	*acrD/yffB*	339	14.57
Esr061	3553578	3553635	*z3927/z3929*	57	2.39
Esr062	3553644	3553801	*z3927/z3929*	157	1.30
Esr063	3665479	3665606	*iap/ygbF*	127	0.57
Esr064	3876048	3876195	*fba/pgk*	147	1.45
Esr065	3878495	3878607	*epd/z4267*	112	0.36
Esr066	4001554	4001692	*Z4403/Z4405*	138	0.11
Esr067	4001926	4002073	*Z4403/Z4405*	147	0.30
Esr068	4009947	4010119	*cca/uppP*	172	0.67
Esr069	4019692	4019893	*dnaG/rpoD*	201	0.34
Esr070	4045213	4045352	*ygjR/ygjT*	139	3.73
Esr071	4114176	4114320	*yhbW/mtr*	144	0.75
Esr072	4432352	4432634	*yhiI/yhiJ*	282	0.51
Esr073	4449216	4449345	*gor/arsR*	129	0.93
Esr074	4449772	4449916	*gor/arsR*	144	0.99
Esr075	4467165	4467606	*hdeD/yhiE*	441	0.27
Esr076	4468310	4468457	*yhiE/yhiU*	147	0.82
Esr077	4475325	4475487	*yhiX/gadA*	162	23.67
Esr078	4600281	4600389	*grxC/yibN*	108	1.03

Esr079	4712818	4713088	*ivbL/emrD*	270	0.40
Esr080	4732779	4732870	*yidB/gyrB*	91	0.34
Esr081	4799589	4799705	*rbsB/rbsK*	116	0.77
Esr082	4864415	4864578	*z5332/corA*	163	0.48
Esr083	4918263	4918470	*hemN/glnG*	207	0.75
Esr084	4921101	4921187	*glnL/glnA*	86	4.43
Esr085	4922784	4922966	*glnA/yihK*	182	0.31
Esr086	5057386	5057514	*rplA/rplJ*	128	1.36
Esr087	5230055	5230122	*ydjF/fumB*	67	0.27
Esr088	5369776	5369982	*holC/pepA*	206	0.29
Esr089	5382157	5382267	*z5883/z5884*	110	0.32
Esr090	5425563	5425814	*yjhA/fimB*	251	0.6
Esr091	5461648	5461794	*yjiO/z5940*	146	0.04
Esr092	2793	2949	*cbp2/L7007*	156	0.34
Esr093	47413	47803	*L7053/L7054*	390	0.47
Esr094	57661	57847	*sopB/L7070*	186	0.39
Esr095	66410	66596	*L7079/L7080*	186	0.28
Esr096	70351	70516	*L7085/L7086*	165	0.02

[a]Fold change of RPKM in O157 Hela-attached and DMEM-grown samples

Supplementary Table S4: List of differentially regulated genes by *Esr055* determined by analysis of transcriptome data

Gene	Description	Fold_change[a]
**213 downregulated genes:**		
Z1192	IS1 protein InsB	-16.83
Z5633	maltose ABC transporter ATP-binding protein	-10.89
Z1632	IS1 protein InsB	-10.14
Z3462	quinol dehydrogenase periplasmic component	-8.04
Z5634	maltoporin	-7.95
Z2983	tail fiber assembly protein of prophage CP-933T	-7.27
Z5632	maltose ABC transporter substrate-binding protein	-6.67
Z0879	hypothetical protein	-6.64
Z0951	exonuclease	-6.55
Z3460	citrate reductase cytochrome C subunit	-6.52
Z5674	formate-dependent nitrite reductase complex subunit NrfF	-6.28
Z4748	hypothetical protein	-5.89
Z1355	hypothetical protein	-5.66
Z5631	maltose transporter membrane protein	-5.35
Z0388	hypothetical protein	-5.19
Z5635	maltose regulon periplasmic protein	-5.13
Z4079	hypothetical protein	-4.93
Z5928	hypothetical protein	-4.57
Z4398	hypothetical protein	-4.55
Z4628	hypothetical protein	-4.54
Z1845	ssDNA-binding protein	-4.42
Z3463	nitrate reductase catalytic subunit	-4.36
Z3365	host-nuclease inhibitor protein Gam of prophage CP-933V	-4.33
Z2977	hypothetical protein	-4.20
Z3461	quinol dehydrogenase membrane component	-4.19
Z1263	hypothetical protein	-4.18
Z5704	phosphonate metabolism protein	-4.13
Z1377	tail component encoded by cryptic prophage CP-933M	-4.10

Z5219	phosphate ABC transporter substrate-binding protein	-4.03
Z1209	hypothetical protein	-3.99
Z2230	30S ribosomal subunit S22	-3.95
Z3452	disulfide oxidoreductase	-3.92
Z4772	maltodextrin phosphorylase	-3.83
Z5217	phosphate transporter permease subunit PtsA	-3.82
Z1379	tail component encoded by cryptic prophage CP-933M; partial	-3.71
Z4302	L-asparaginase II	-3.49
Z4617	hypothetical protein	-3.36
Z5648	phage shock protein G	-3.36
Z2562	transposase (partial)	-3.34
Z2091	hypothetical protein	-3.33
Z5578	zinc resistance protein	-3.32
Z1452	hypothetical protein	-3.28
Z3712	detox protein	-3.26
Z2729	hypothetical protein	-3.25
Z6072	hypothetical protein	-3.24
Z5803	PTS system L-ascorbate-specific transporter subunit IIB	-3.23
Z5671	formate-dependent nitrite reductase; Fe-S centers	-3.16
Z1003	molybdopterin synthase small subunit	-3.08
Z5218	phosphate transporter permease subunit PstC	-3.07
Z3459	cytochrome C	-3.03
Z4771	4-alpha-glucanotransferase	-3.02
Z2873	hypothetical protein	-2.99
Z2235	formate dehydrogenase-N, nitrate-inducible, iron-sulfur beta subunit	-2.95
Z4001	recombination regulator RecX	-2.94
Z5936	hypothetical protein	-2.92
Z0876	succinate dehydrogenase cytochrome b556 small membrane subunit	-2.90
Z4224	oxidoreductase, Fe-S subunit	-2.87
Z3762	phosphoribosylaminoimidazole synthetase	-2.83
Z5432	hypothetical protein	-2.80
Z0432	cytosine permease	-2.74
Z1547	acyl carrier protein	-2.73
Z4191	type III secretion apparatus protein	-2.73
Z5663	hypothetical protein	-2.72
Z1431	hypothetical protein	-2.71
Z3575	colicin V production protein	-2.66

Z0678	phosphoribosylaminoimidazole carboxylase catalytic subunit	-2.66
Z0534	cytochrome o ubiquinol oxidase subunit I	-2.66
Z3323	hypothetical protein	-2.65
Z0533	cytochrome o ubiquinol oxidase subunit III	-2.65
Z5082	transporter	-2.64
L7058	hypothetical protein	-2.63
Z3404	galactose/methyl galaxtoside transporter ATP-binding protein	-2.62
Z3451	subunit of heme lyase	-2.61
Z0532	cytochrome o ubiquinol oxidase subunit IV	-2.61
Z2551	tryptophan synthase subunit alpha	-2.61
Z4871	nickel transporter ATP-binding protein NikD	-2.59
Z2550	tryptophan synthase subunit beta	-2.59
Z4169	hypothetical protein	-2.59
Z3413	lysine transporter	-2.59
Z3465	ferredoxin-type protein	-2.58
Z0677	phosphoribosylaminoimidazole carboxylase ATPase subunit	-2.57
Z3763	phosphoribosylglycinamide formyltransferase	-2.57
Z4156	diaminopimelate decarboxylase	-2.55
Z1211	adhesin	-2.48
Z1651	adhesin	-2.48
Z0636	transcriptional regulator	-2.43
Z2554	hypothetical protein	-2.42
Z5583	bifunctional phosphoribosylaminoimidazolecarboxamide formyltransferase/IMP cyclohydrolase	-2.41
Z3458	cytochrome c biogenesis protein CcmA	-2.40
Z5215	transcriptional regulator PhoU	-2.39
Z0877	succinate dehydrogenase flavoprotein subunit	-2.38
Z4072	phosphoadenosine phosphosulfate reductase	-2.38
Z5007	hypothetical protein	-2.35
Z4240	glycine dehydrogenase	-2.31
Z4870	nickel transporter permease NikC	-2.31
Z1649	hypothetical protein	-2.28
Z3405	galactose-binding transport protein; receptor for galactose taxis	-2.28
L7030	hypothetical protein	-2.28
Z5166	ilvB operon leader peptide	-2.28
Z2201	fimbrial chaperone protein	-2.27
Z0001	thr operon leader peptide	-2.27

Z2236	formate dehydrogenase-N, nitrate-inducible, alpha subunit	-2.26
Z1992	cation transport regulator	-2.26
Z5164	acetolactate synthase 1 regulatory subunit	-2.25
Z0961	endopeptidase Rz of prophage CP-933K	-2.25
Z3835	phosphoribosylformylglycinamidine synthase	-2.24
Z1663	hypothetical protein	-2.24
Z1910	hypothetical protein	-2.24
Z0498	phosphate regulon sensor protein	-2.24
Z0433	cytosine deaminase	-2.23
Z3781	nucleoside diphosphate kinase	-2.22
Z4369	outer membrane lipoprotein	-2.22
Z6040	head-tail adaptor of cryptic prophage CP-933P	-2.22
Z0662	hydroxypyruvate isomerase	-2.22
Z3403	beta-methylgalactoside transporter inner membrane protein	-2.21
Z3464	assembly protein for periplasmic nitrate reductase	-2.21
Z0296	hypothetical protein	-2.20
Z4931	cytochrome C peroxidase	-2.20
Z0878	succinate dehydrogenase iron-sulfur subunit	-2.20
Z1308	SOS cell division inhibitor	-2.20
Z0969	hypothetical protein	-2.19
Z4367	hypothetical protein	-2.19
Z1433	hypothetical protein	-2.18
Z1851	hypothetical protein	-2.16
Z0981	prophage protein	-2.16
Z5669	cytochrome c552	-2.15
Z3909	recombination and repair protein	-2.14
Z0890	hypothetical protein	-2.14
Z1088	hypothetical protein	-2.14
Z3574	amidophosphoribosyltransferase	-2.13
Z1366	hypothetical protein	-2.13
Z3802	hydrolase	-2.13
Z5630	maltose ABC transporter permease	-2.10
Z1332	hypothetical protein	-2.09
Z0531	protoheme IX farnesyltransferase	-2.09
Z1852	holin protein of prophage CP-933C	-2.09
Z2162	hypothetical protein	-2.09
Z4959	dipeptide transporter	-2.08
Z0512	nucleoside channel phage T6/colicin K receptor	-2.08
Z4747	hypothetical protein	-2.07

Z4942	C4-dicarboxylate transporter DctA	-2.06
Z1777	hypothetical protein	-2.06
Z4171	hypothetical protein	-2.06
Z1634	hypothetical protein	-2.05
Z2678	superoxide dismutase	-2.05
Z3760	uracil transporter	-2.05
Z0037	carbamoyl phosphate synthase small subunit	-2.05
Z2058	hypothetical protein	-2.05
Z2200	major fimbrial subunit	-2.05
Z0535	cytochrome o ubiquinol oxidase subunit II	-2.04
Z3440	hypothetical protein	-2.04
Z2479	DNA-binding transcriptional activator PspC	-2.04
Z4047	decarboxylase	-2.04
Z3313	tail component of prophage CP-933V	-2.04
Z5700	phosphonate C-P lyase system protein PhnK	-2.03
Z1983	N5-glutamine S-adenosyl-L-methionine-dependent methyltransferase	-2.02
Z4955	hypothetical protein	-2.02
Z5920	mannonate dehydratase	-2.01
Z3453	cytochrome C biogenesis protein	-2.01
Z2590	transporter	-2.00
Z0960	lysozyme protein R of prophage CP-933K	-2.00
Z1038	hypothetical protein	>-100
L7004	putative hemolysin expression-modulating protein	>-100
L7009	replication protein	>-100
L7053	putative serine-threonine protein kinase	>-100
Z0344	hypothetical protein	>-100
Z0416	ABC transporter ATP-binding protein	>-100
Z0653	hypothetical protein	>-100
Z1151	hypothetical protein	>-100
Z1180	hypothetical protein	>-100
Z1191	hypothetical protein	>-100
Z1326	inhibitor of cell division encoded by cryptic prophage CP-933M	>-100
Z1350	holin protein of cryptic prophage CP-933M	>-100
Z1405	cold shock-like protein	>-100
Z1588	hypothetical protein	>-100
Z1590	hypothetical protein	>-100
Z1620	hypothetical protein	>-100
Z1624	hypothetical protein	>-100

Z1805	hypothetical protein	>-100
Z1839	hypothetical protein	>-100
Z1856	hypothetical protein	>-100
Z1926	hypothetical protein	>-100
Z1960	hypothetical protein	>-100
Z1967	hypothetical protein	>-100
Z2046	DNA-binding transcriptional regulator DicC	>-100
Z2068	hypothetical protein	>-100
Z2086	division inhibition protein DicB within CP-933O	>-100
Z2106	hypothetical protein	>-100
Z2231	malate dehydrogenase	>-100
Z2254	H repeat-containing Rhs element protein	>-100
Z2293	hypothetical protein	>-100
Z2310	hypothetical protein	>-100
Z2343	outer membrane protein Lom encoded by prophage CP-933R	>-100
Z2370	hypothetical protein	>-100
Z2393	hypothetical protein	>-100
Z2872	hypothetical protein	>-100
Z2972	hypothetical protein	>-100
Z2976	hypothetical protein	>-100
Z3166	hypothetical protein	>-100
Z3327	hypothetical protein	>-100
Z3336	endopeptidase Rz for prophage CP-933V	>-100
Z3368	hypothetical protein	>-100
Z3641	hypothetical protein	>-100
Z4037	hydrogenase assembly chaperone	>-100
Z4062	hypothetical protein	>-100
Z4192	hypothetical protein	>-100
Z4325	hypothetical protein	>-100
Z4982	small toxic polypeptide	>-100
Z5093	hypothetical protein	>-100
Z5095	hypothetical protein	>-100
Z5199	hypothetical protein	>-100
Z5791	hypothetical protein	>-100
Z6043	hypothetical protein	>-100

205 upregulated genes:

Z2232	alcohol dehydrogenase	82.61
Z3167	hypothetical protein	9.49
Z2804	hypothetical protein	7.50
Z5339	hypothetical protein	7.45

Z3307	tail fiber protein encoded within prophage CP-933V	5.49
Z1347	hypothetical protein	5.46
Z4045	hypothetical protein	5.33
Z4035	hydrogenase nickel incorporation protein	4.73
Z4461	hypothetical protein	4.17
Z5952	hypothetical protein	3.94
Z0248	hypothetical protein	3.89
Z1889	DNA packaging protein of prophage CP-933X	3.87
Z5337	hypothetical protein	3.83
Z1574	complement resistance protein	3.71
Z2975	hypothetical protein	3.70
Z2136	major capsid protein of prophage CP-933O	3.63
Z2318	phosphatidate cytidylyltransferase	3.62
Z0336	regulatory protein encoded in prophage CP-933I	3.61
Z3596	minor fimbrial subunit	3.58
Z0690	fimbrial asembly protein	3.51
Z3118	hypothetical protein	3.51
Z1123	hypothetical protein	3.38
Z0313	hypothetical protein	3.26
Z1713	flagellar basal body rod modification protein	3.19
Z2059	hypothetical protein	3.16
Z0328	hypothetical protein	3.14
Z0354	ferredoxin	3.13
Z1197	hypothetical protein	3.07
Z5667	hypothetical protein	2.98
Z2759	hypothetical protein	2.95
Z2533	hypothetical protein	2.92
Z5018	hypothetical protein	2.91
Z1098	hypothetical protein	2.91
Z3257	PTS system galactitol-specific transporter subunit IIA	2.91
Z5022	hypothetical protein	2.85
Z1944	hemolysin E	2.85
Z4471	DNA-binding transcriptional activator TdcR	2.83
Z2493	hypothetical protein	2.83
Z2980	stability/partitioning protein encoded within CP-933T	2.80
Z1711	flagellar basal-body rod protein FlgB	2.79
Z0050	ferredoxin	2.76
Z0392	hypothetical protein	2.75
Z5028	hypothetical protein	2.74

Z0332	activator encoded in prophage CP-933I	2.73
Z2175	hypothetical protein	2.73
Z2390	hypothetical protein	2.71
Z3121	hypothetical protein	2.69
Z3043	hypothetical protein	2.68
Z3597	minor fimbrial subunit	2.67
Z2274	hypothetical protein	2.64
Z3718	hypothetical protein	2.64
Z2374	holin protein of prophage CP-933R	2.63
Z0485	hypothetical protein	2.63
Z4198	regulatory protein for type III secretion apparatus	2.62
Z2265	transferase	2.61
Z5729	hypothetical protein	2.61
Z3222	colanic acid biosynthesis acetyltransferase WcaB	2.59
Z4077	hypothetical protein	2.59
Z0245	hypothetical protein	2.58
L7078	hypothetical protein	2.57
Z3322	major tail subunit encoded within prophage CP-933V	2.56
Z4180	lipoprotein of type III secretion apparatus	2.56
Z0869	chaperone	2.56
Z1717	flagellar basal body L-ring protein	2.54
Z3215	GDP-mannose mannosyl hydrolase	2.53
Z4181	Type III secretion apparatus protein	2.51
Z1795	hypothetical protein	2.48
Z1714	flagellar hook protein FlgE	2.43
Z4282	hypothetical protein	2.41
Z3704	hypothetical protein	2.38
Z2233	hypothetical protein	2.38
Z3022	hypothetical protein	2.37
Z5713	proline/glycine betaine transporter	2.37
Z0898	hypothetical protein	2.37
Z1465	shiga-like toxin II B subunit encoded by bacteriophage BP-933W	2.36
Z5509	fructose-like phosphotransferase EIIB subunit 3	2.36
Z0333	polarity suppression protein encoded in CP-933I	2.36
Z1677	autoagglutination protein	2.35
Z0404	hypothetical protein	2.35
Z1877	endopeptidase of prophage CP-933X	2.34
Z5445	hypothetical protein	2.33
Z1155	hypothetical protein	2.33
Z4802	ATP-dependent DNA helicase	2.33

Z0746	hypothetical protein	2.32
Z5131	hypothetical protein	2.32
Z5917	fimbrial morphology protein	2.31
Z5224	fimbrial chaperone	2.29
Z5945	endoribonuclease SymE	2.29
Z0311	hypothetical protein	2.29
Z0691	fimbrial protein	2.27
Z2713	hypothetical protein	2.27
Z0757	triphosphoribosyl-dephospho-CoA synthase	2.25
Z5121	hypothetical protein	2.25
Z3064	sulfite oxidase subunit YedZ	2.25
Z0688	chaperone	2.25
Z1556	hypothetical protein	2.24
Z4844	sulfur transfer protein SirA	2.24
Z2685	inner membrane transport protein YdhC	2.23
Z1509	hypothetical protein	2.23
Z1708	flagellar synthesis protein FlgN	2.23
Z2560	hypothetical protein	2.23
Z5914	periplasmic chaperone, required for type 1 fimbriae	2.21
Z1552	hypothetical protein	2.21
Z1534	chaperone	2.21
Z5940	hypothetical protein	2.20
Z4324	transposase	2.20
Z1807	hypothetical protein	2.19
Z3219	glycosyl transferase	2.19
L7096	putative transposase	2.19
Z1927	hypothetical protein	2.18
Z1772	hypothetical protein	2.15
Z1426	hypothetical protein	2.14
Z4443	hypothetical protein	2.14
Z1027	hypothetical protein	2.13
Z4108	hypothetical protein	2.13
Z2074	IS encoded protein within CP-933O	2.13
Z1855	hypothetical protein	2.12
Z4439	hypothetical protein	2.09
Z1553	hypothetical protein	2.07
Z0271	hypothetical protein	2.07
Z5954	hypothetical protein	2.06
Z1507	hypothetical protein	2.06
Z0568	hypothetical protein	2.05
Z0409	oxidoreductase subunit	2.05

Z5160	hypothetical protein	2.05
Z5644	stress-response protein	2.05
Z3961	gamma-aminobutyrate transporter	2.04
Z3154	hypothetical protein	2.04
Z1622	hypothetical protein	2.04
Z5187	hypothetical protein	2.04
Z0351	hypothetical protein	2.03
Z0977	tail component of prophage CP-933K	2.02
Z4335	hypothetical protein	2.00
L7051	hypothetical protein	>100
Z0028	hypothetical protein	>100
Z0039	hypothetical protein	>100
Z0274	hypothetical protein	>100
Z0362	hypothetical protein	>100
Z0380	hypothetical protein	>100
Z0389	hypothetical protein	>100
Z0406	hypothetical protein	>100
Z0664	hypothetical protein	>100
Z0761	citrate lyase subunit gamma	>100
Z0968	hypothetical protein	>100
Z0974	tail component of prophage CP-933K	>100
Z1125	hypothetical protein	>100
Z1136	hypothetical protein	>100
Z1137	hypothetical protein	>100
Z1225	hypothetical protein	>100
Z1356	hypothetical protein	>100
Z1408	cold shock gene	>100
Z1557	hypothetical protein	>100
Z1564	hypothetical protein	>100
Z1575	hypothetical protein	>100
Z1712	flagellar basal body rod protein FlgC	>100
Z1867	integrase of prophage CP-933X	>100
Z1912	hypothetical protein	>100
Z2042	hypothetical protein	>100
Z2055	hypothetical protein	>100
Z2105	hypothetical protein	>100
Z2122	holin protein of prophage CP-933O	>100
Z2181	hypothetical protein	>100
Z2229	biofilm-dependent modulation protein	>100
Z2261	Rhs element protein	>100
Z2283	hypothetical protein	>100
Z2406	FtsZ inhibitor protein	>100

Z2413	hypothetical protein	>100
Z2414	hypothetical protein	>100
Z2566	integrase fragment, cryptic prophage CP-933P	>100
Z2631	oriC-binding nucleoid-associated protein	>100
Z2658	hypothetical protein	>100
Z2828	hypothetical protein	>100
Z2838	hypothetical protein	>100
Z2973	hypothetical protein	>100
Z2988	tail fiber protein component of prophage CP-933T	>100
Z3024	hypothetical protein	>100
Z3073	hypothetical protein	>100
Z3090	hypothetical protein	>100
Z3163	hypothetical protein	>100
Z3165	hypothetical protein	>100
Z3316	hypothetical protein	>100
Z3319	hypothetical protein	>100
Z3334	hypothetical protein	>100
Z3359	hypothetical protein	>100
Z3621	hypothetical protein	>100
Z3811	3-phenylpropionate dioxygenase ferredoxin subunit	>100
Z3843	hypothetical protein	>100
Z3941	hypothetical protein	>100
Z4071	hypothetical protein	>100
Z4090	hypothetical protein	>100
Z4188	type III secretion apparatus protein	>100
Z4286	hypothetical protein	>100
Z4322	hypothetical protein	>100
Z4775	hypothetical protein	>100
Z5213	hypothetical protein	>100
Z5431	hypothetical protein	>100
Z5694	hypothetical protein	>100
Z5731	hypothetical protein	>100
Z5754	entericidin B	>100
Z5859	hypothetical protein	>100
Z5904	hypothetical protein	>100
Z6011	hypothetical protein	>100
Z6035	tail assembly protein of cryptic prophage CP-933P	>100
Z6068	hypothetical protein	>100

Z6078	inhibitor of cell division encoded by cryptic prophage CP-933P	>100

[a]Fold change of RPKM in O157 wild-type and Δ*Esr055* samples; -, downregulated

Supplementary Table S5: Confirmation of RNA-seq data by qRT-PCR

**Gene**	**Fold change**		**Description**
	**RNA-Seq**	**Real-time PCR[a]**	
z0690	3.51	4.92	fimbrial asembly protein
z1465	2.36	4.14	shiga-like toxin II B subunit encoded by bacteriophage BP-933W
z2804	7.50	8.76	hypothetical protein
z3222	2.59	3.19	colanic acid biosynthesis acetyltransferase WcaB
z4802	2.33	4.20	ATP-dependent DNA helicase
z0191	1.03	0.89	UDP-3-O-[3-hydroxymyristoyl] glucosamine N-acyltransferase
z2284	0.85	1.44	collagenase
z3586	1.01	1.03	3-oxoacyl-ACP synthase
z5052	0.92	1.23	lipopolysaccharide core biosynthesis protein
z6001	1.17	0.91	DNA-binding response regulator CreB
z0535	-2.04	-2.5	cytochrome o ubiquinol oxidase subunit II
z1992	-2.26	-3.13	cation transport regulator
z3413	-2.59	-3.85	lysine transporter
z4959	-2.08	-3.23	dipeptide transporter
z5674	-6.28	-8.33	formate-dependent nitrite reductase complex subunit NrfF
z0688	2.25	4.77	fimbrial chaperone
z0691	2.27	5.32	fimbrial protein
z1708	2.23	8.94	flagellar synthesis protein FlgN
z1711	2.79	4.38	flagellar basal-body rod protein FlgB
z1712	>100	7.61	flagellar basal body rod protein FlgC
z1713	3.19	6.77	flagellar basal body rod modification protein
z1714	2.43	6.92	flagellar hook protein FlgE
z1717	2.54	5.51	flagellar basal body L-ring protein
z3596	3.58	2.38	minor fimbrial subunit
z3597	2.69	2.62	minor fimbrial subunit
z5914	2.12	6.42	periplasmic chaperone, required for type 1 fimbriae
z5917	2.31	2.05	fimbrial morphology protein

[a]Data presented are the mean values from three independent experiments.

References

Datsenko, K.A., and Wanner, B.L. (2000). One-step inactivation of chromosomal genes in *Escherichia coli* K-12 using PCR products. *Proc Natl Acad Sci U S A* 97(12)**,** 6640-6645. doi: 10.1073/pnas.120163297.

Duan, K., Dammel, C., Stein, J., Rabin, H., and Surette, M.G. (2003). Modulation of *Pseudomonas aeruginosa* gene expression by host microflora through interspecies communication. *Mol Microbiol* 50(5)**,** 1477-1491.

Ju, H., Zou, R., Venema, V.J., and Venema, R.C. (1997). Direct interaction of endothelial nitric-oxide synthase and caveolin-1 inhibits synthase activity. *J Biol Chem* 272(30)**,** 18522-18525.

Wang, R.F., and Kushner, S.R. (1991). Construction of versatile low-copy-number vectors for cloning, sequencing and gene expression in *Escherichia coli*. *Gene* 100**,** 195-199.
